# Tolerability, safety, and pharmacokinetics of a single intravenous administration of a novel recombinant humanized anti-interleukin-6 receptor monoclonal antibody in healthy Chinese volunteers

**DOI:** 10.3389/fphar.2023.1267178

**Published:** 2024-01-31

**Authors:** Xin Jiang, Pingping Lin, Feifei Sun, Yi Xu, Ye Tao, Ping Shi, Yanping Liu, Xin Li, Shuqin Liu, Xiaomeng Gao, Chenjing Wang, Yu Cao

**Affiliations:** Phase I Clinical Research Center, The Affiliated Hospital of Qingdao University, Qingdao, China

**Keywords:** interleukin-6 receptor, safety, tolerability, pharmacokinetics, healthy volunteers

## Abstract

**Aim:** VDJ001 is a novel recombinant humanized monoclonal antibody against the anti-interleukin-6 receptor. As an analog of tocilizumab, it exhibited improved affinity and *in vitro* activity. Based on preclinical studies, a first-in-human clinical study was conducted to evaluate the safety, tolerability, and pharmacokinetics of VDJ001.

**Methods:** This is a single-center, randomized, double-blinded, placebo-controlled phase I dose-escalation study conducted in healthy Chinese volunteers. Four cohorts were designed with dosages ranging from 1 to 8 mg/kg. There were equal numbers of female and male volunteers in each cohort. Enrolled subjects randomly received a single intravenous administration of VDJ001 or placebo (VDJ001: placebo = 4:1 in both female and male volunteers). Three sentinel volunteers in the 1 mg/kg cohort were first administered, and the treatment of the other seven volunteers was carried out after a safety assessment on D15. The following cohort was conducted only when the safety profile was evaluated as acceptable on D29 of the previous cohort. Samples for pharmacokinetics (PK), pharmacodynamics (PD), and immunogenicity were collected at specified time points and analyzed through validated methods. Adverse events and the results of the examination and laboratory were analyzed to assess the safety profile.

**Results:** All cohorts were carried out according to the protocol. With the escalation of dosage, C_max_ increased linearly, and AUC_0-t_ and AUC_0-∞_ increased in a non-linear manner, while clearance decreased and t1/2 prolonged. Six volunteers who received VDJ001 tested ADA-positive, among whom one participant tested Nab-positive on D57. One volunteer in the placebo group tested ADA-positive but Nab-negative. CRP concentrations were not found to be correlated with the dosage. Both IL-6 and sIL-6R concentrations increased after the administration of VDJ001. All adverse events were mild to moderate in severity. No serious adverse events were reported in this study. No unexpected or clinically significant safety issues were found.

**Conclusion:** The safety and tolerability of VDJ001 are acceptable with a single intravenous dosage of 1∼8 mg/kg. Further clinical trials are warranted.

## 1 Introduction

Interleukin-6 (IL-6), a cytokine synthesized by diverse types of cells, plays significant roles in multiple physiological processes such as inflammation, immune response, hematopoiesis, bone metabolism, apoptosis, and differentiation or proliferation of some nonimmune cells (Tanaka et al., 2018; Kaur et al., 2020; Uciechowski and Dempke, 2020). Due to its pleiotropic feature, excessive production of IL-6 is involved in a variety of chronic inflammation, and autoimmune and other diseases (Tanaka et al., 2014). IL-6 signaling pathway blockage was considered a potential strategy to develop novel therapeutics for the aforementioned diseases, and great efforts made in this field have been rewarded with the approval of several novel drugs in the last decades (Choy et al., 2020). Tocilizumab (TCZ) is a first-in-class recombinant humanized monoclonal antibody against the IL-6 receptor (IL-6R), which has been marketed as a treatment for Castleman’s disease, moderate to severe active rheumatoid arthritis (RA) in adults, and juvenile idiopathic arthritis (Sheppard et al., 2017).

TCZ is relatively expensive, and in some cases, there may be supply shortages (Gombolay and Hallman-Cooper, 2023), which limit the use of TCZ by some patients. Developing biologics with the same mechanism of action to give patients more choices is of great significance. By modifying the amino acid sequences of TCZ, a novel recombinant humanized anti-IL6R monoclonal antibody codenamed VDJ001 was developed. Using a Biacore system, the affinity of VDJ001 for IL-6R was determined to be approximately 1000-fold higher than that of TCZ. Its *in vitro* activity evaluated by DS-1 cells was more than 10 times greater than that of TCZ (detailed information on preclinical studies will be published later). Based on preclinical studies of VDJ001, a first-in-human clinical study was conducted.

## 2 Materials and methods

### 2.1 Study design

A single-center, randomized, double-blind, placebo controlled, phase I dose-escalation clinical trial was designed to study the tolerability, safety, and pharmacokinetics of VDJ001 in healthy Chinese volunteers. There were four preset dose levels in this study: 1, 2, 4, and 8 mg/kg. VDJ001 and placebo were supplied as injections. Each cohort enrolled five male and five female volunteers, and randomization stratified by gender was performed. Participants randomly received a single intravenous infusion of VDJ001 or placebo (4:1 in both strata). For safety consideration, three sentinel volunteers were first administered VDJ001 or placebo in the starting cohort, and then, after a safety assessment on D15, the treatment of the other seven volunteers was conducted. On D29 of each cohort, a safety assessment was carried out to determine whether the next higher dose group was to initiate.

On the day before administration (D-1), enrolled volunteers were admitted to the study site. One hour before dinner on D-1, the body weights of volunteers were measured to calculate their dosage. VDJ001 or placebo was diluted with 0.9% saline into 100 mL and intravenously infused with infusion pumps for at least 60 min on D1. Volunteers were discharged on D4 and returned to the study site for outpatient visits on D5, D6, D8, D11, D15, D22, D29, D43 (not applicable to the 1 and 2 mg/kg dose groups), and D57. At scheduled time points, blood samples for pharmacokinetics (PK), pharmacodynamics (PD), and immunogenicity were collected, and safety assessments were conducted.

The study was approved by the Ethics Committee of the Affiliated Hospital of Qingdao University (QYFYEC 2019-042-01) and registered on chinadrugtrials.org.cn (CTR20191199.19 June 2019) and clinicaltrials.gov (NCT06021574). The study was conducted in accordance with the Declaration of Helsinki, Good Clinical Practice principles, and applicable laws and regulations. All volunteers provided written informed consent prior to being screened for eligibility.

### 2.2 Subjects

This study enrolled 40 healthy Chinese volunteers, with an equal distribution of male and female volunteers. Eligible volunteers should meet all the inclusion and exclusion criteria of the study protocol (see Supplementary Material).

### 2.3 Pharmacokinetic analysis

PK samples were collected prior to infusion (within 1 h) and at 0, 2, 4, 8, 12, 24 (D2), 48 (D3), 72 (D4), 96 (D5), 120 (D6), 168 (D8), 240 (D11), 336 (D15), 504 (D22), and 672 h (D29), following infusion for all cohorts. For the 4-mg/kg and 8-mg/kg dose groups, additional samples were collected at 1008 (D43) and 1344 h (D57). After standing for at least 30 min, blood samples were centrifuged and serum samples were frozen until transferred to be analyzed.

The serum concentrations of VDJ001 were determined by a validated enzyme-linked immunosorbent assay (ELISA) method. The linear range of the standard curve was 150–4,800 ng/mL. The lower and upper limits of quantification were 150 ng/mL and 4800 ng/mL, respectively. Pharmacokinetic parameters include the area under the concentration–time curve (AUC) from administration to the last measurable concentration (AUC_0-t_), AUC extrapolated to infinity (AUC_0-∞_), maximum serum concentration (C_max_), time to C_max_ (T_max_), elimination half-life (t_1/2_), volume of distribution (V_z_), clearance rate (CL_z_), elimination rate constant (λ_z_), mean residence time (MRT) from administration to the last measurable concentration (MRT_0-t_), and MRT extrapolated to infinity (MRT_0-∞_) were calculated by a non-compartment model using Phoenix WinNonlin 8.1 (Certara, Princeton, NJ, United States of America).

### 2.4 Immunogenicity assessment

Samples for immunogenicity analysis were taken pre-dose (within 1 h) and at 336 (D15), 504 (D22), 672 (D29), and 1,344 h (D57) post-dose for all dose levels. Concentrations of anti-drug antibodies (ADAs) in serum were measured by a validated bridging-ELISA method to assess the immunogenicity of VDJ001. Neutralizing antibodies (Nab) were detected when necessary.

### 2.5 Pharmacodynamic assessment

Concentrations of C-reactive protein (CRP), IL-6, and sIL-6R were quantified to assess PD of VDJ001. Blood samples for the CRP assessment were obtained before dosing (within 1 h) and at 2, 24 (D2), 72 (D4), 336 (D15), 672 (D29), and 1344 h (D57) post-dose for all cohorts. Samples for IL-6 and sIL-6R assessment were collected prior to administration of VDJ001 or placebo (within 1 h) and at 2, 24 (D2), 72 (D4), 168 (D8), 336 (D15), 672 (D29), and 1344 h (D57), following administration for all cohorts. The serum concentrations of IL-6 were analyzed using a validated ELISA method. The linear range of the standard curve was 0.400–10.0 pg/mL. Serum sIL-6R levels were determined by a validated ELISA with a linear range of 10.0–640 ng/mL.

Immunogenicity and PD samples were processed and stored under the same conditions as PK samples.

### 2.6 Statistical analysis

Statistical analyses were performed by SAS 9.4 (SAS Institute Inc., Cary, NC, United States) in this study. Continuous variables were described using number of cases, mean, standard deviation, quartiles, minimum, and maximum values, while count and grade data were presented as frequency and percentage. During PK analysis, values below the quantitation limit before and after T_max_ were seen as “0” and missing, respectively.

### 2.7 Safety assessments

Safety was evaluated using data on adverse events (AEs), vital signs (including blood pressure, heart rate, and body temperature), physical examination, laboratory tests, and 12-lead electrocardiograms.

## 3 Results

### 3.1 Study population

Four cohorts were all carried out, and further escalation was not continued after discussion. Overall, 206 subjects were screened, and 40 were enrolled in this study, with 10 in each group. The quantity of male volunteers equals that of female volunteers. All enrolled subjects completed the procedures required according to the protocol. Demographic data on the enrolled subjects are shown in Table 1.

**TABLE 1:** Demographic data on the enrolled subjects.

	1 mg/kg (N = 8)	2 mg/kg (N = 8)	4 mg/kg (N = 8)	8 mg/kg (N = 8)	Overall (N = 32)	Placebo (N = 8)
Age^a^ (year)	22.00 (19.00, 42.00)	33.00 (19.00, 44.00)	23.00 (19.00, 38.00)	28.50 (22.00, 38.00)	25.00 (19.00,44.00)	31.0 0(22.00, 37.00)
Height^b^ (cm)	168.75 (7.13)	165.75 (11.24)	167.88 (7.84)	166.31 (9.14)	167.17 (8.61)	161.81 (6.96)
Body weight^b^ (kg)	63.06 (5.17)	60.44 (9.13)	62.69 (8.61)	61.56 (9.10)	61.94 (7.84)	59.88 (5.20)
BMI^b^ (kg/m^2^)	22.22 (1.51)	22.05 (1.71)	22.16(1.78)	22.53 (2.11)	22.24 (1.71)	22.93 (1.80)

The numbers are presented as follows:

^a^ median (min, max).

^b^ mean (SD). SD, standard deviation.

### 3.2 Pharmacokinetics

**FIGURE 1:**
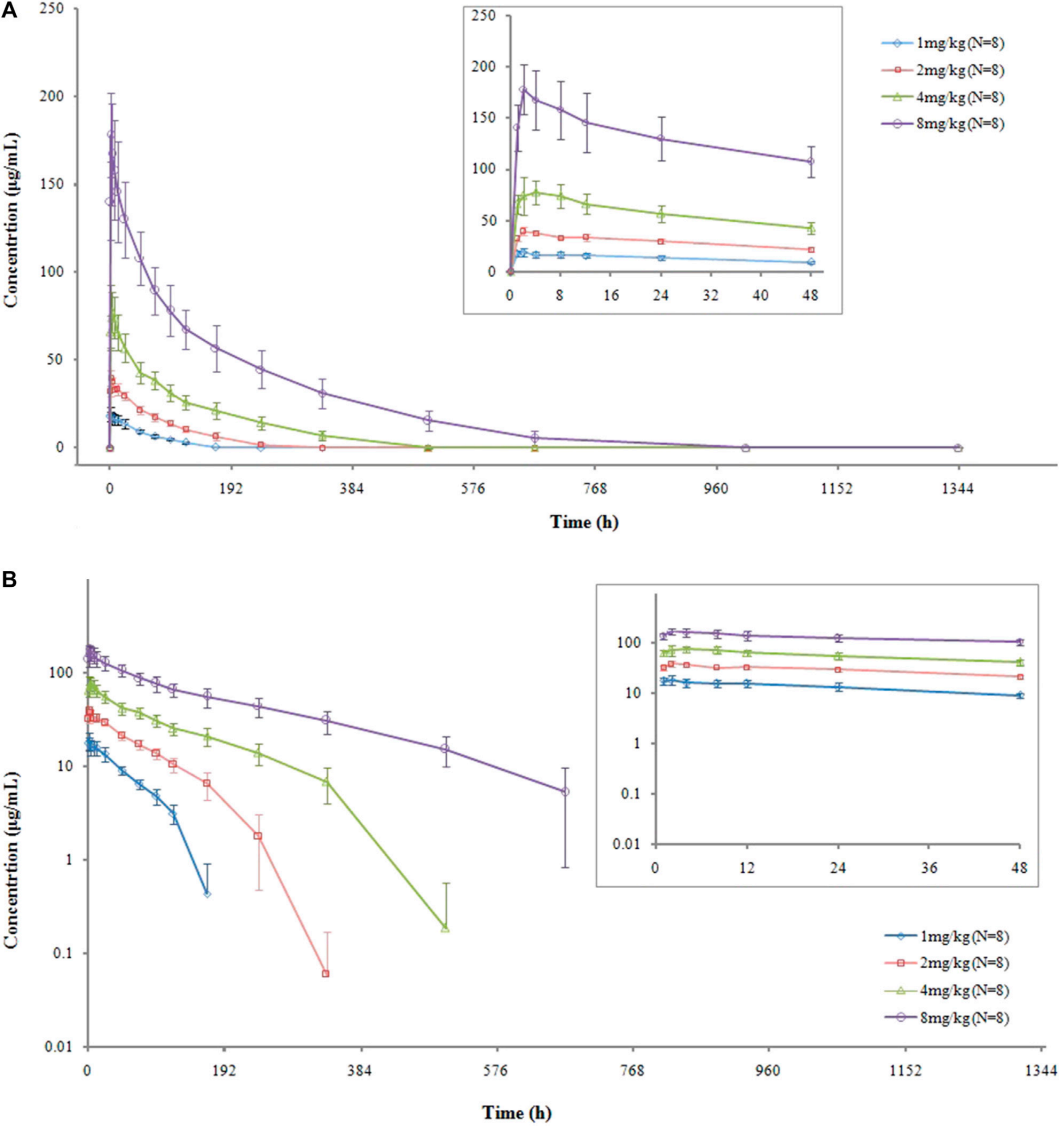
Mean (SD) concentration–time curves of VDJ001: **(A)** linear and **(B)** semilog.

The serum concentration-versus-time curves are shown in Figure 1. After a single intravenous administration of VDJ001, C_max_ at each dose level increased in proportion to the administered dose, which is a linear characteristic. AUC_0-t_ and AUC_0-∞_ also increased with the escalation of dosage but displayed a non-linear feature as the rising range was higher than the dose-proportional relationship. Clearance (CL) decreased when the dose escalated. t_1/2_ prolonged with the increase in dose. There were no significant differences in exposure between genders in each dose group. The main PK parameters are presented in Table 2.

**TABLE 2:** PK parameters of VDJ001.

	1 mg/kg (N = 8)	2 mg/kg (N = 8)	4 mg/kg (N = 8)	8 mg/kg (N = 8)
C_max_ (μg/mL)	20.01 (3.99)	39.79 (3.76)	82.93 (11.37)	181.00 (24.48)
AUC_0-t_(h*μg/mL)	1,141.72 (200.63)	3,322.94 (487.60)	8,993.23 (1736.82)	28,475.44 (5,668.11)
AUC_0-∞_(h*μg/mL)	1,240.48 (153.51)	3,428.00 (434.61)	9,646.96 (1,628.53)	30,002.53 (6,756.07)
T_max_ (h)	3.53 (2.51)	3.50 (0.72)	5.00 (2.72)	4.52 (2.12)
t_1/2_ (h)	38.22 (8.57)	46.97 (11.21)	92.60 (19.57)	153.82 (42.28)
V_z_ (mL/kg)	46.18 (13.84)	40.37 (11.07)	57.08 (15.34)	59.65 (10.19)
CL_z_ (mL/h/kg)	0.82 (0.10)	0.59 (0.08)	0.43 (0.08)	0.28 (0.06)
λ_z_ (×10^-3^ 1/h)	19.18 (5.40)	15.62 (4.24)	7.80 (1.76)	4.73 (0.98)
MRT_0-t_ (h)	46.64 (5.23)	72.01 (11.10)	113.03 (14.95)	183.07 (20.70)
MRT_0-∞_ (h)	58.95 (4.99)	79.47 (8.82)	139.63 (14.68)	216.43 (47.01)

PK, parameters are shown as mean (SD).

### 3.3 Immunogenicity

The ADA positive rate for subjects administered VDJ001 was 18.8% (6/32). In the 1-mg/kg group, no subject was ADA-positive. In the 2, 4, and 8 mg/kg groups, there were three subjects, two subjects, and one subject who were ADA-positive, respectively. Among these ADA-positive subjects, one from the 2-mg/kg group tested positive for Nab. In the placebo group, one female subject was ADA-positive but Nab-negative.

### 3.4 Pharmacodynamics

**FIGURE 2:**
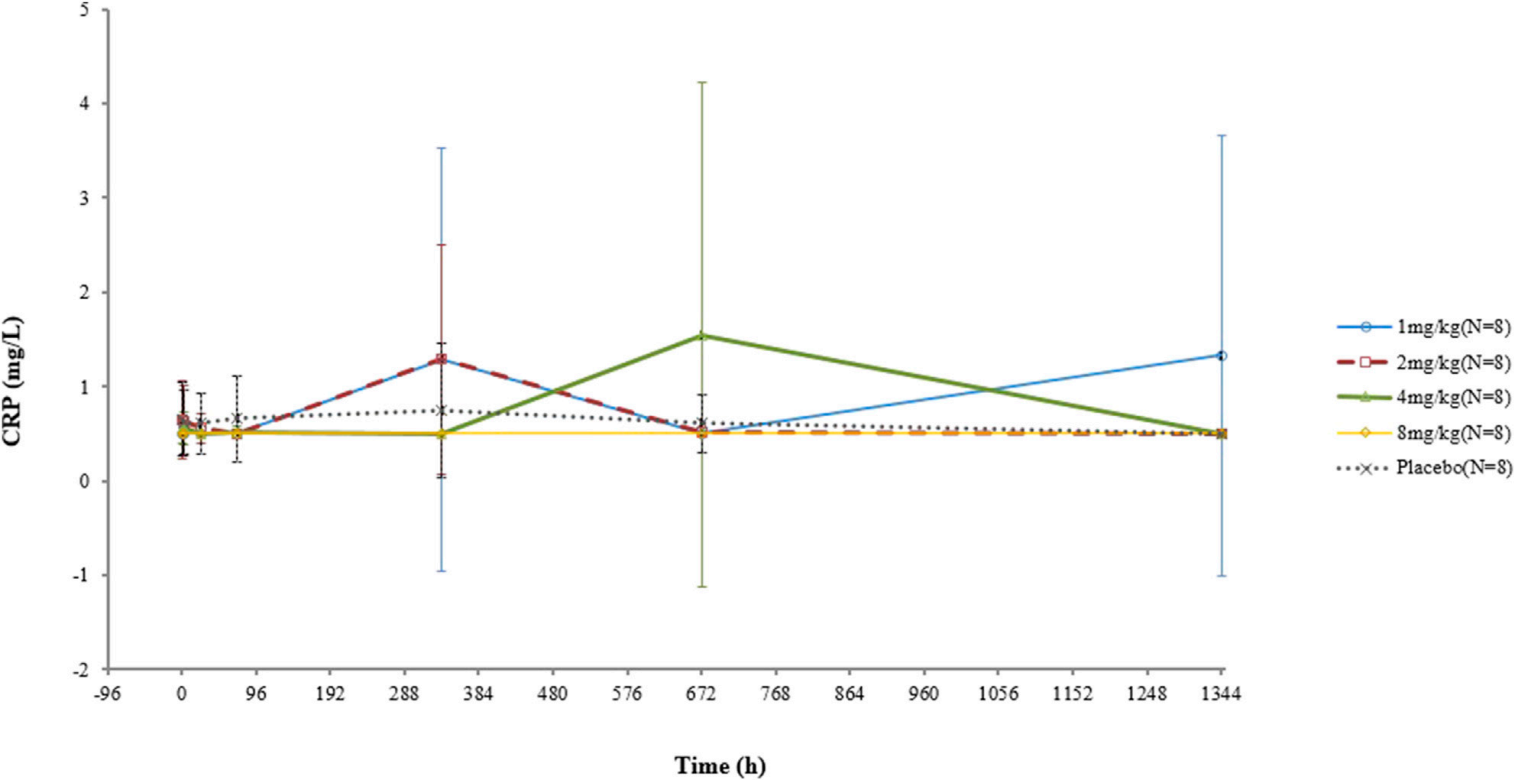
Mean (SD) CRP concentration–time curve after a single dose of VDJ001.

The CRP concentrations of two subjects who received a 1-mg dosage were elevated at D15 and D57, respectively, both of which became normal in subsequent visits. One participant in the 4-mg/kg group had a high CRP level at D29 before returning to normal. No increase in the CRP concentration was observed in the other subjects. No significant correlation between the CRP concentration and dose was found (Figure 2).

**FIGURE 3:**
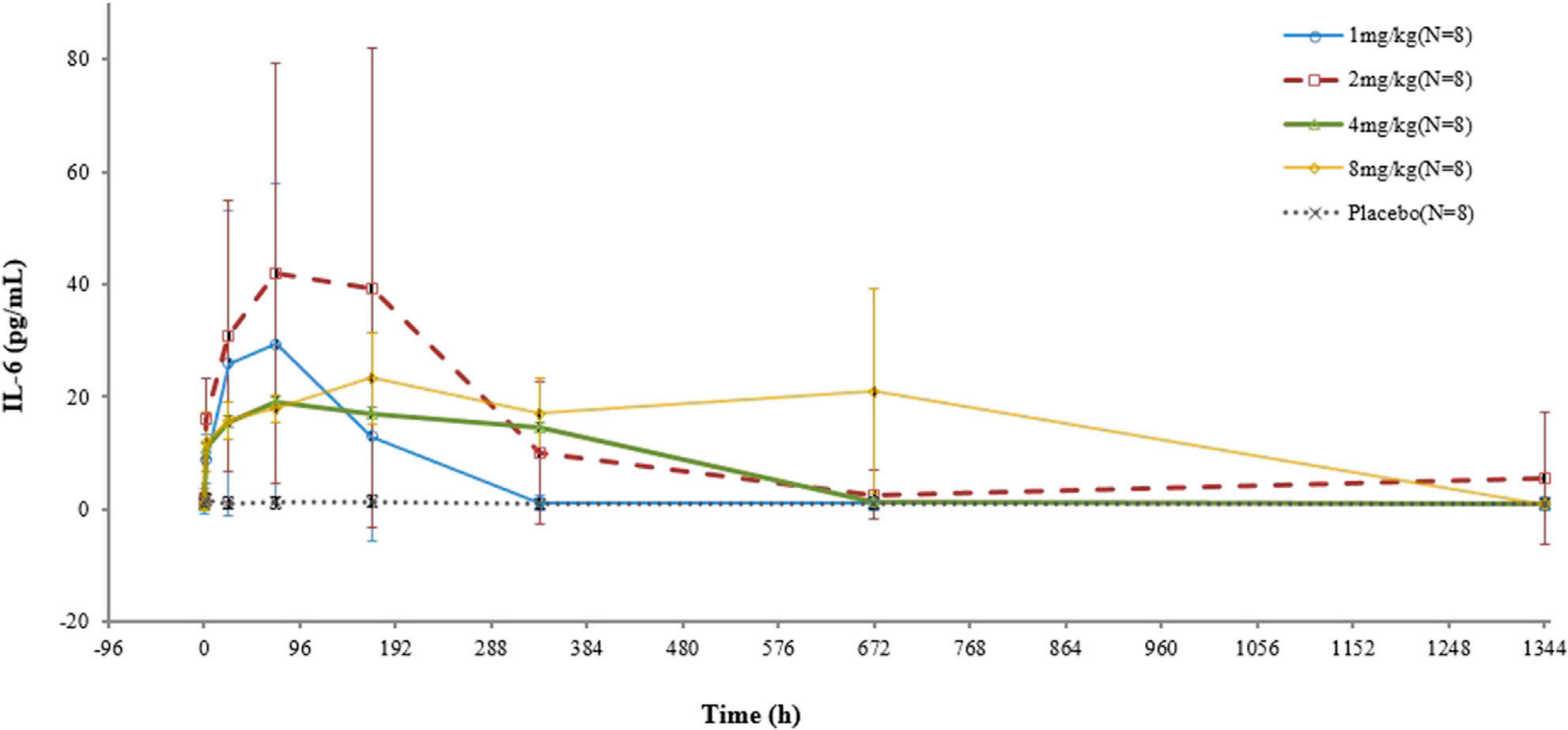
Mean (SD) concentration–time profile of IL-6 after a single intravenous administration of VDJ001.

In the test group, IL-6 concentrations increased after infusion, which was not correlated with the dose. On the other hand, in the placebo group, there was no substantial change in the IL-6 concentration (Figure 3).

**FIGURE 4:**
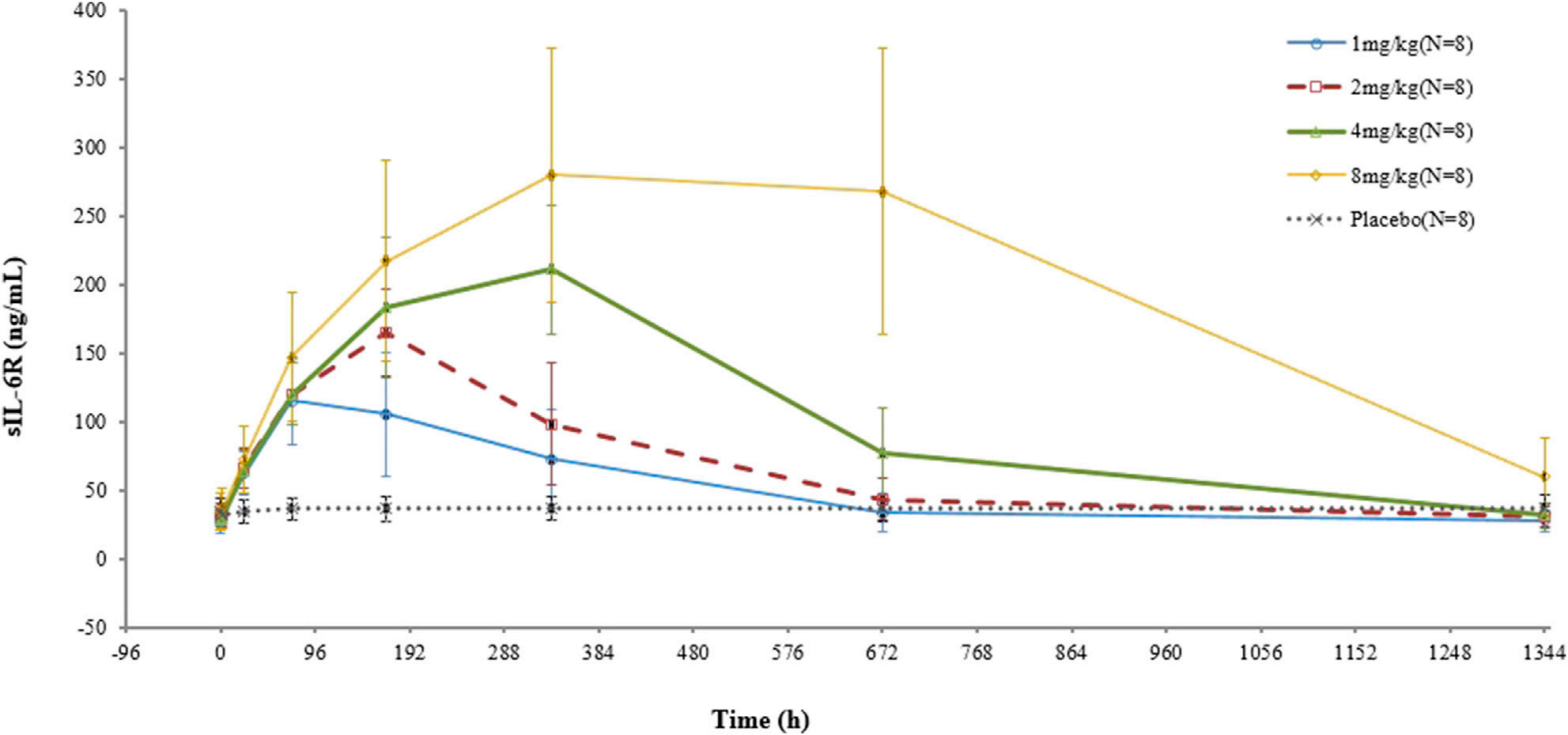
Mean (SD) sIL-6R concentration–time curve after administration of a single dose of VDJ001.

In terms of sIL-6R, higher concentrations were observed in all test groups, and the higher the dose, the greater the increase. No significant change was found in the placebo group (Figure 4).

### 3.5 Safety

Totally, there were 69 AEs reported by 32 subjects in this study: 61 AEs were reported by 27 subjects in the test group, and 8 AEs were reported by 5 subjects in the placebo group (Table 3). Among all AEs, two AEs from two subjects were deemed not treatment-related AEs. No SAEs and AEs resulting in the withdrawal of subjects were reported. The escalation of doses was not terminated due to safety issues.

**TABLE 3:** Summary of AEs.

AE	1 mg/kg (N = 8)		2 mg/kg (N = 8)		4 mg/kg (N = 8)		8 mg/kg (N = 8)		Overall (N = 32)		Placebo (N = 8)	
	n(%)	nAEs^*^	n(%)	nAEs^*^	n(%)	nAEs^*^	n(%)	nAEs^*^	n(%)	nAEs^*^	n(%)	nAEs^*^
**Total**	6(75.0)	15	7(87.5)	15	8(100.0)	15	6(75.0)	16	27(84.4)	61	5(62.5)	8
**Investigation**	5(62.5)	9	7(87.5)	9	7(87.5)	9	5(62.5)	11	24(75.0)	38	3(37.5)	4
Blood triglycerides increased	0(0)	0	1(12.5)	1	1(12.5)	1	2(25.0)	4	4(12.5)	6	0(0)	0
Neutrophil count decreased	0(0)	0	2(25.0)	2	1(12.5)	1	1(12.5)	1	4(12.5)	4	0(0)	0
White blood cell count increased	0(0)	0	0(0)	0	2(25.0)	2	1(12.5)	1	3(9.4)	3	0(0)	0
Alanine aminotransferase increased	0(0)	0	0(0)	0	0(0)	0	2(25.0)	3	2(6.3)	3	1(12.5)	1
Antinuclear antibody positive	1(12.5)	1	0(0)	0	2(25.0)	2	0(0)	0	3(9.4)	3	0(0)	0
Transaminases increased	2(25.0)	2	0(0)	0	1(12.5)	1	0(0)	0	3(9.4)	3	0(0)	0
White blood cells in urine (positive)	1(12.5)	1	0(0)	0	0(0)	0	0(0)	0	1(3.1)	1	1(12.5)	1
Urine ketone body presence	0(0)	0	1(12.5)	1	0(0)	0	0(0)	0	1(3.1)	1	1(12.5)	1
Aspartate aminotransferase increased	0(0)	0	1(12.5)	1	0(0)	0	1(12.5)	1	2(6.3)	2	0(0)	0
QT interval prolonged	1(12.5)	1	1(12.5)	1	0(0)	0	0(0)	0	2(6.3)	2	0(0)	0
Blood bilirubin increased	1(12.5)	1	0(0)	0	0(0)	0	0(0)	0	1(3.1)	1	1(12.5)	1
Fibrinogen decreased	0(0)	0	0(0)	0	1(12.5)	1	1(12.5)	1	2(6.3)	2	0(0)	0
C-reactive protein increased	1(12.5)	1	0(0)	0	0(0)	0	0(0)	0	1(3.1)	1	0(0)	0
White blood cell count decreased	0(0)	0	1(12.5)	1	0(0)	0	0(0)	0	1(3.1)	1	0(0)	0
Urinary sediment present	0(0)	0	1(12.5)	1	0(0)	0	0(0)	0	1(3.1)	1	0(0)	0
Crystal urine	0(0)	0	0(0)	0	1(12.5)	1	0(0)	0	1(3.1)	1	0(0)	0
Heart rate increased	1(12.5)	1	0(0)	0	0(0)	0	0(0)	0	1(3.1)	1	0(0)	0
Blood potassium decreased	1(12.5)	1	0(0)	0	0(0)	0	0(0)	0	1(3.1)	1	0(0)	0
Blood sodium decreased	0(0)	0	1(12.5)	1	0(0)	0	0(0)	0	1(3.1)	1	0(0)	0
**Infections and infestations**	2(25.0)	2	1(12.5)	1	3(37.5)	4	1(12.5)	1	7(21.9)	8	0(0)	0
Upper respiratory tract infections	0(0)	0	1(12.5)	1	2(25.0)	2	0(0)	0	3(9.4)	3	0(0)	0
Nasopharyngitis	1(12.5)	1	0(0)	0	0(0)	0	0(0)	0	1(3.1)	1	0(0)	0
Urinary tract infection	0(0)	0	0(0)	0	0(0)	0	1(12.5)	1	1(3.1)	1	0(0)	0
Gastroenteritis	1(12.5)	1	0(0)	0	0(0)	0	0(0)	0	1(3.1)	1	0(0)	0
Pulpitis	0(0)	0	0(0)	0	1(12.5)	1	0(0)	0	1(3.1)	1	0(0)	0
Pharyngitis	0(0)	0	0(0)	0	1(12.5)	1	0(0)	0	1(3.1)	1	0(0)	0
**Gastrointestinal disorders**	2(25.0)	2	0(0)	0	0(0)	0	1(12.5)	1	3(9.4)	3	3(37.5)	3
Diarrhea	2(25.0)	2	0(0)	0	0(0)	0	0(0)	0	2(6.3)	2	1(12.5)	1
Mouth ulcer	0(0)	0	0(0)	0	0(0)	0	0(0)	0	0(0)	0	2(25.0)	2
Noninfective gingivitis	0(0)	0	0(0)	0	0(0)	0	1(12.5)	1	1(3.1)	1	0(0)	0
**Blood and lymphatic system disorders**	1(12.5)	1	1(12.5)	1	1(12.5)	1	1(12.5)	1	4(12.5)	4	1(12.5)	1
Anemia	1(12.5)	1	1(12.5)	1	0(0)	0	0(0)	0	2(6.3)	2	1(12.5)	1
Neutrophilia	0(0)	0	0(0)	0	1(12.5)	1	1(12.5)	1	2(6.3)	2	0(0)	0
**Hepatobiliary disorders**	0(0)	0	1(12.5)	1	0(0)	0	1(12.5)	1	2(6.3)	2	0(0)	0
Function liver abnormal	0(0)	0	0(0)	0	0(0)	0	1(12.5)	1	1(3.1)	1	0(0)	0
Liver injury	0(0)	0	1(12.5)	1	0(0)	0	0(0)	0	1(3.1)	1	0(0)	0
**Skin and subcutaneous tissue disorders**	0(0)	0	1(12.5)	1	1(12.5)	1	0(0)	0	2(6.3)	2	0(0)	0
Dermatitis	0(0)	0	1(12.5)	1	0(0)	0	0(0)	0	1(3.1)	1	0(0)	0
Eczema	0(0)	0	0(0)	0	1(12.5)	1	0(0)	0	1(3.1)	1	0(0)	0
**Nervous system disorders**	0(0)	0	1(12.5)	1	0(0)	0	0(0)	0	1(3.1)	1	0(0)	0
Headache	0(0)	0	1(12.5)	1	0(0)	0	0(0)	0	1(3.1)	1	0(0)	0
**Injury, poisoning, and procedural complications**	0(0)	0	1(12.5)	1	0(0)	0	0(0)	0	1(3.1)	1	0(0)	0
Limb injury	0(0)	0	1(12.5)	1	0(0)	0	0(0)	0	1(3.1)	1	0(0)	0
**Musculoskeletal and connective tissue disorders**	1(12.5)	1	0(0)	0	0(0)	0	0(0)	0	1(3.1)	1	0(0)	0
Myalgia	1(12.5)	1	0(0)	0	0(0)	0	0(0)	0	1(3.1)	1	0(0)	0
**Renal and urinary disorders**	0(0)	0	0(0)	0	0(0)	0	1(12.5)	1	1(3.1)	1	0(0)	0
Calculus urinary	0(0)	0	0(0)	0	0(0)	0	1(12.5)	1	1(3.1)	1	0(0)	0

## 4 Discussion

This is the first-in-human phase I dose escalation study of VDJ001 to evaluate its safety, tolerability, PK profiles, and immunogenicity. The no-observed adverse effect level (NOAEL) of VDJ001 in the cynomolgus monkey was 10 mg/kg. The human equivalent dose (HED) was determined to be 10 mg/kg based on NOAEL. Divided by a safety factor of 10, the calculated maximum recommended safe dose was 1 mg/kg. Considering the dose of TCZ in healthy volunteers (CLINICAL PHARMACOLOGY AND BIOPHARMACEUTICS REVIEWS, 2023), 1 mg/kg was chosen as the initial dose. Dose escalation would be terminated when a) ≥ grade 2 adverse events occurred in half or more volunteers or ≥ grade 3 adverse events occurred in at least one-third of the volunteers in one cohort; b) serious adverse events related to investigational drugs occurred; or c) grade 4 infusion-related reactions (IRRs), or grade 3 IRRs ineffective to corticosteroid treatment, which occurred within 24 h after the start of administration. The 8-mg/kg cohort did not meet the criteria to terminate dose escalation. However, the results of these preset cohorts met expectations. To avoid unnecessary exposure in healthy volunteers, further dose escalation was not conducted.

In the preset dose range of 1 mg/kg to 8 mg/kg, VDJ001 exhibited an acceptable safety profile in healthy volunteers. All AEs reported in this study were grade 1 or 2 based on Common Terminology Criteria for Adverse Events (CTCAEs) 5.0. The incidences of AEs in the test and placebo groups were 84.4% and 62.5%, respectively. The most common AEs that occurred in the test group were laboratory findings (75.0%), followed by infections and infestations (21.9%). In the placebo group, the most common AEs were laboratory findings (37.5%) and gastrointestinal disorders (37.5%). Close attention should be paid to the aforementioned AEs in subsequent clinical trials. The incidence of AEs did not increase significantly with the increase in dose.

After a single administration of the test preparation within the dose level of 1∼8 mg/kg, C_max_ increased dose proportionally, while AUC_0-t_ and AUC_0-∞_ increased more than the dose-proportional level. In preclinical PK studies conducted in cynomolgus monkeys, a single dose of VDJ001 exhibited non-linear PK characteristics in the range of 1 mg/kg to 50 mg/kg. C_max_ increases proportionally with dose, while AUC_0-t_ and AUC_0-∞_ increase greater than the dose-proportional level. Similar non-linear PK profiles also appeared after four consecutive doses of VDJ001, from 1 mg/kg to 100 mg/kg once a week. This non-linear PK profile may result from the change in clearance pathways when the dose of VDJ001 increases as compared to other monoclonal antibodies (Dirks and Meibohm, 2010). At a low dose of VDJ001, it is mainly eliminated by the receptor-mediated clearance pathway. With the increase in dose, this pathway becomes saturated, and non-specific Fc-receptor-mediated elimination becomes the main clearance pathway (Dirks and Meibohm, 2010). During this process, the clearance of VDJ001 reduces, t_1/2_ prolongs, and AUC increases.

The IL-6 concentration is determined by the production and elimination rates of IL-6. IL-6 is eliminated by catabolism and target-mediated clearance, with the latter being predominant. The production and catabolism of IL-6 are relatively constant (Nishimoto et al., 2008). When the dose of VDJ001 increased from 1 mg/kg to 8 mg/kg, IL-6R became fully occupied, and IL-6R-mediated elimination was blocked. This may be the reason why there was no clear correlation between the IL-6 level and dose.

In the test group, six subjects tested ADA-positive, of whom only one subject tested Nab-positive at D57 (16.7%). In the placebo group, one subject was observed ADA-positive before treatment. The ADA test can be carried out during screening or pre-dose in subsequent clinical trials to distinguish false positives. To evaluate the effect of ADA on PK of VDJ001, mean serum concentrations and primary PK parameters, including C_max_, AUC_0-t_, and AUC_0-∞_ of ADA-positive and ADA-negative subjects, were compared in each cohort. There were no significant differences between the results of the ADA-positive and ADA-negative groups in the 2-mg/kg and 4-mg/kg cohorts. PK data were not affected by ADA in these two cohorts. In the 8-mg/kg cohort, the differences were significant. The reason for these differences may be that only one subject was ADA-positive in this cohort. The effect of ADA on PK needs to be studied further if clinical studies are carried out at this dose level in the future.

Three minor deviations from protocol were reported in this study, all of which were reviewed by the ethics committee. The safety of the subjects and the outcomes of this study were not affected by these deviations.

At present, only one monoclonal antibody directly blocking IL-6, namely, siltuximab, has received approval, and other antibodies with the same mechanism of action have not been approved due to safety issues in clinical trials or ongoing clinical studies (McElvaney et al., 2021). Antibodies targeting IL-6R displayed a high level of safety profile compared to IL-6-targeting antibodies, and tocilizumab and sarilumab of this class have been approved (Chen et al., 2021). VDJ001 is a novel monoclonal antibody inhibiting IL-6R. It showed effects better than or equivalent to tocilizumab in preclinical studies, and its safety and tolerability were proved in this study, which makes it a promising therapy for treating IL-6 signaling pathway-related diseases.

In preclinical pharmacokinetic studies, the effect of 4 mg/kg VDJ001 on reducing CRP was comparable to that of 12 mg/kg tocilizumab. In the collagen-induced arthritis model, VDJ001 at 4 mg/kg showed a comparable effect to 12 mg/kg of tocilizumab in terms of changing clinical scores, body weight, and joint swelling. In this study, the safety after administration of 1–8 mg/kg VDJ001 is acceptable. The effects of VDJ001 on sIL-6R and IL-6 were similar to those of tocilizumab (Nishimoto et al., 2008). It is expected that doses of 4–6 mg/kg can match the performance of tocilizumab and are suitable for further development.

## 5 Conclusion

A single intravenous administration of VDJ001 at a dose level of 1–8 mg/kg is safe and well-tolerated. Subsequent clinical studies in patients can be carried out within this dose range.

## Data Availability

The data that support the reported results are available on the request from the corresponding author. The data are publicly unavailable due to privacy or ethical restrictions.
