# Supplementary material for: Tolerability, safety, and pharmacokinetics of a single intravenous administration of a novel recombinant humanized anti-interleukin-6 receptor monoclonal antibody in healthy Chinese volunteers
Source: Front Pharmacol. 2024 Jan 31;14:1267178. doi: 10.3389/fphar.2023.1267178 (PMC10864494; doi:10.3389/fphar.2023.1267178)
Supplement: Supplementary file 1 [file Table1.DOCX]

Inclusion criteria included:

(1) Sign the informed consent form and comply with the protocol to complete the study.

(2) Age ≥18 years old and ≤45 years old (at the date of signing the informed consent); females weigh between 45.0~75.0kg, males weigh between 50.0~80.0kg, the body mass index ( BMI) within the range of 19.0~26.0kg/m^2^.

(3) Function of heart, liver and kidney is normal.

(4) The blood pregnancy test for female of childbearing age is negative, and the volunteers or their spouses use appropriate and effective contraceptive measures, such as abstinence, intrauterine device and double barrier method from the test period to 6 months after administration of study drugs.

Exclusion criteria were as follows:

(1) Allergic constitution (e.g. allergic to two or more drugs / foods).

(2) Those who have a clear history of malignant tumors, neurological or mental disorders (including epilepsy or dementia), heart failure, or immunodeficiency or immunosuppressive diseases, or have undergone major surgery within 6 months before randomization.

(3) Those with severe bleeding factors that affect venous blood collection or those who are unable/unwilling to receive venipuncture.

(4) Those who have received any vaccines within 6 months before randomization.

(5) Those who have previously received anti-IL-6 drugs or other monoclonal antibody treatments, or have used any biological agents within 12 months before randomization.

(6) Those who have administered with other clinical trial drugs within 3 months before randomization.

(7) Massive bleeding or whole blood or component blood (> 400ml) donation within 3 months before randomization.

(8) With active bacterial, viral, fungal, or parasitic infection or any infection that requires systemic anti-infective treatment within 4 weeks before randomization.

(9) Those who have received any health products or treated by drugs (including prescription drugs such as warfarin, cyclosporine, theophylline, simvastatin, omeprazole, dextromethorphan, over-the-counter drugs, proprietary Chinese medicine, vitamins, dietary supplements, etc.) within 3 months before randomization or those who may receive drugs during the trial.

(10) Those who have used soft drugs (such as marijuana) within 3 months before randomization or hard drugs (such as cocaine, phencyclidine, etc.) within 1 year before the trial; or those with positive drug abuse test results (cocaine, marijuana, morphine, amphetamine, methamphetamine, phencyclidine, benzodiazepines, barbiturates, methadone and tricyclic antidepressants).

(11) Alcoholics or those who drink regularly within 3 months before randomization, or those with a positive alcohol breath test.

(12) Those who smoke within 1 month before screening.

(13) Those with positive results of nicotine test during screening.

(14) Drinking excessive amounts of tea, coffee and/or caffeinated beverages every day.

(15) Positive results of γ interferon release test (T-SPOT).

(16) Positive result for any one of human immunodeficiency virus (HIV) antibody, treponema pallidum antibody, hepatitis C virus antibody, hepatitis B surface antigen.

(17) Result of rheumatoid factor, anti-double-stranded DNA antibody, antinuclear antibody, or anti-cyclic citrulline antibody test is abnormal with clinical significance.

(18) Those who plan to undergo surgery (including cosmetic surgery, dental surgery and oral surgery) during the trial period; or those who plan to participate in strenuous exercise (including physical contact exercise or collision exercise) during the trial period.

(19) Those who schedule to donate blood during the trial period; or plan to receive any biological drugs within 3 months after the administration of study drug or those who plan to receive monoclonal antibody drugs within 9 months after the administration of study drug.

(20) Those who plan to get pregnant, or to donate sperm or eggs within 6 months after the administration of study drugs.

(21) Not suitable for enrollment or may not be able to complete the trial due to other reasons determined by investigator.

(22) For females: be pregnant or lactating; had unprotected sex with their partner within 14 days before the trial; have taken oral contraceptives 30 days before the trial or plan to take them during the trial; or have used or plan to use long-acting estrogen or progesterone injections or implants within 6 months before the trial.
